# Improved Lung Function With Cyclophosphamide and Rituximab in a Case of Shrinking Lung Syndrome

**DOI:** 10.7759/cureus.97612

**Published:** 2025-11-23

**Authors:** Shiza Chaudhry, Haider Bokhary, Mark Quinn

**Affiliations:** 1 Internal Medicine, York and Scarborough Teaching Hospitals NHS Foundation Trust, York, GBR; 2 Respiratory Medicine, Pinderfields Hospital, Wakefield, GBR; 3 Rheumatology, York and Scarborough Teaching Hospitals NHS Foundation Trust, York, GBR

**Keywords:** dyspnea, restrictive lung disease, rheumatology, shrinking lung syndrome, systemic lupus erythematosus

## Abstract

Shrinking lung syndrome (SLS) is a rare respiratory complication of systemic autoimmune diseases. It is most commonly associated with systemic lupus erythematosus (SLE), often presenting as a diagnostic challenge. It is characterized by hallmark clinical features of dyspnea and pleuritic chest pain, along with radiographic evidence of reduced lung volumes and restrictive lung physiology in the absence of interstitial lung disease. Patients often demonstrate positive antibody serology due to associated underlying autoimmune conditions. In particular, anti-nuclear antibodies (ANA), low complement levels, and, in certain instances, extractable nuclear antigens (ENA) tend to be positive. We present a case of a young lady in her 20s who developed exertional shortness of breath, pleuritic chest pain, and reduced exercise tolerance. In the context of a positive autoimmune profile suggestive of SLE, typical radiological features, and pulmonary function tests, she was diagnosed with shrinking lung syndrome (SLS). The case describes therapeutic strategies that exist for the treatment of SLS, which can bring about meaningful recovery in lung function. The overall prognosis of SLS is variable, but outcomes may be favorable with timely recognition and appropriate treatment.

## Introduction

Dyspnea is a common symptom associated with systemic autoimmune diseases, such as systemic lupus erythematosus (SLE). Some more common causes of dyspnea in these patients include pleural disease, pneumonitis, pulmonary hypertension, and interstitial lung disease [1]. Nonetheless, some patients experience unexplained dyspnea despite comprehensive evaluation.

Among the rarer pulmonary manifestations of autoimmune disease is shrinking lung syndrome (SLS). The condition was first described by Hoffbrand and Beck in 1965 [2]. This is most commonly seen in patients with SLE, with an estimated prevalence of 0.5-1.53% in patients with this condition [3]. However, it can also be seen in other autoimmune conditions, such as rheumatoid arthritis, systemic sclerosis, and Sjogren’s disease. There are several theories regarding the etiology of SLS, including diaphragmatic dysfunction, pleural inflammation, and phrenic neuropathy. However, there is no clear evidence to explain the exact pathophysiology of this condition so far [3].

The most common symptoms seen in SLS are exertional dyspnea (99%) and pleuritic chest pain (76%). Fever (19%) and dry cough (8%) are rarely seen in patients with SLS. Patients can have concomitant features of SLE, most commonly arthritis (77%). Tachypnea, reduced chest wall expansion, and use of accessory muscles of respiration are the most frequently seen features on clinical examination. It is common to see a diagnostic delay in this condition, with 48% of patients presenting with over a year of symptoms before being formally diagnosed [4]. Here, we report a case of a young lady with early-onset SLS in the setting of SLE who demonstrated sustained response to combined cyclophosphamide and rituximab therapy.

## Case presentation

A young lady in her 20s was under the care of the rheumatology department for inflammatory arthropathy. She initially presented with arthralgias, particularly of the small joints of the hands and feet. On examination, there was an absence of synovitis with normal cardiorespiratory, neurological, and abdominal examinations. Of note, there were no oral ulcers, skin rashes, or pericardial or pleural rub. Her autoimmune profile was positive for anti-nuclear antibodies (ANA) (homogeneous at 1/640), anti-RNP, anti-Smith, anti-dsDNA, and anti-Ro60 antibodies with normal complement levels. At the time of initial presentation, she did not meet the European Alliance of Associations for Rheumatology (EULAR)/American College of Rheumatology (ACR) diagnostic criteria for SLE, scoring 6/12 points for positive anti-dsDNA/anti-Smith antibodies [5]. Joint involvement was not significant enough to be counted towards the score. As such, she was treated for an articular presentation of possible mixed connective tissue disease (MCTD) with reducing dose of prednisolone (starting at 30 mg once daily, reducing by 5 mg every four days), hydroxychloroquine 200 mg twice daily, and oral methotrexate 20 mg once a week.

Several months following her diagnosis of MCTD, she developed exertional dyspnea, pleuritic chest pain, and reduced exercise tolerance. She had been diagnosed with right basal pneumonia on a recent hospital admission and was subsequently treated with multiple courses of oral antibiotics. However, she showed minimal improvement. Interval imaging showed full resolution of the right basal pneumonia, and CT pulmonary angiography showed no evidence of pulmonary embolism.

Three months after treatment of her pneumonia, she remained symptomatic with persistent shortness of breath on minimal exertion, fatigue, and infrequent pleuritic chest pain brought on by coughing. It was noted that around the same time, her joint symptoms significantly worsened. She had developed swelling of her hands and feet, along with morning stiffness that lasted more than an hour. At a follow-up clinic appointment, joint examination revealed polyarticular swelling and stiffness, particularly involving the small joints of her hands and feet, as well as left ankle swelling. On systemic examination, she was also noted to have decreased air entry in both lung bases on auscultation. Neurologically, she had 5/5 power, with normal tone, reflexes, coordination, and sensations in both upper and lower limbs. Cranial nerves were intact. She had unremarkable cardiac and abdominal examinations. A full set of investigations was performed, including full blood count, biochemistry, repeat autoimmune serology, inflammatory markers, and urinalysis. Results of the relevant investigations are detailed in Table 1 below.

**Table 1:** Results of relevant investigations, including full blood count, biochemistry, autoimmune serology, and urinalysis. CRP: C-reactive protein; ESR: erythrocyte sedimentation rate; anti-dsDNA: anti-double-stranded DNA; B2-glycoprotein IgG: beta-2 glycoprotein immunoglobulin G; ANCA: anti-neutrophil cytoplasmic antibodies; anti-CCP: anti-cyclic citrullinated antibody; anti-RNP: anti-ribonucleoprotein; C3: complement component; C4: complement component; eGFR: estimated glomerular filtration rate

Tests	Patient’s result	Reference range
Full blood count		
Hemoglobin	129 g/L	115-165 g/L
White blood cell count	3.8x10^9^/L	4.0-11.0x10^9^/L
Neutrophils	3.1x10^9^/L	2.0-8.0x10^9^/L
Lymphocytes	0.4x10^9^/L	0.5-4.5x10^9^/L
Monocytes	0.1x10^9^/L	0.2-1.2x10^9^/L
Platelet count	374x10^9^/L	150-450x10^9^/L
Mean cell volume	88 fL	79-101 fL
Inflammatory markers		
CRP	46 mg/L	0-5 mg/L
ESR	88 mm/h	1-15 mm/h
Autoimmune serology		
Anti-nuclear antibodies	Homogeneous 1/640	-
Anti-dsDNA	172.8 IU/L	0.000-17.300 IU/L
Anti-cardiolipin	14.7 CU	0.0-20.0 CU
B2-glycoprotein IgG	9.7 CU	0.0-20.0 CU
ANCA	Negative	-
Anti-CCP	Negative	-
Extractable nuclear antigens (ENA)		
Ro60	Positive	-
Ro52	Negative	-
Anti-RNP	Positive	-
Anti-Smith	Positive	-
Jo1	Negative	-
Scl70	Negative	-
Complement		
C4	0.18 g/L	0.10-0.40 g/L
C3	1.52 g/L	0.90-1.80 g/L
Immunoglobulins		
Immunoglobulin A	2.96 g/L	0.80-2.80 g/L
Immunoglobulin M	1.72 g/L	0.50-1.90 g/L
Immunoglobulin G	21.9 g/L	6.0-16.0 g/L
Urinalysis		
Protein	Negative	-
Biochemistry		
Sodium	141 mmol/L	133-146 mmol/L
Potassium	4.0 mmol/L	3.5-5.3 mmol/L
Creatinine	60 μmol/L	45-84 μmol/L
eGFR	120 mL/min/1.73 m^2^	Normal >90 mL/min/1.73 m^2^
Urea	3.9 mmol/L	2.5-7.8 mmol/L

**Figure 1:**
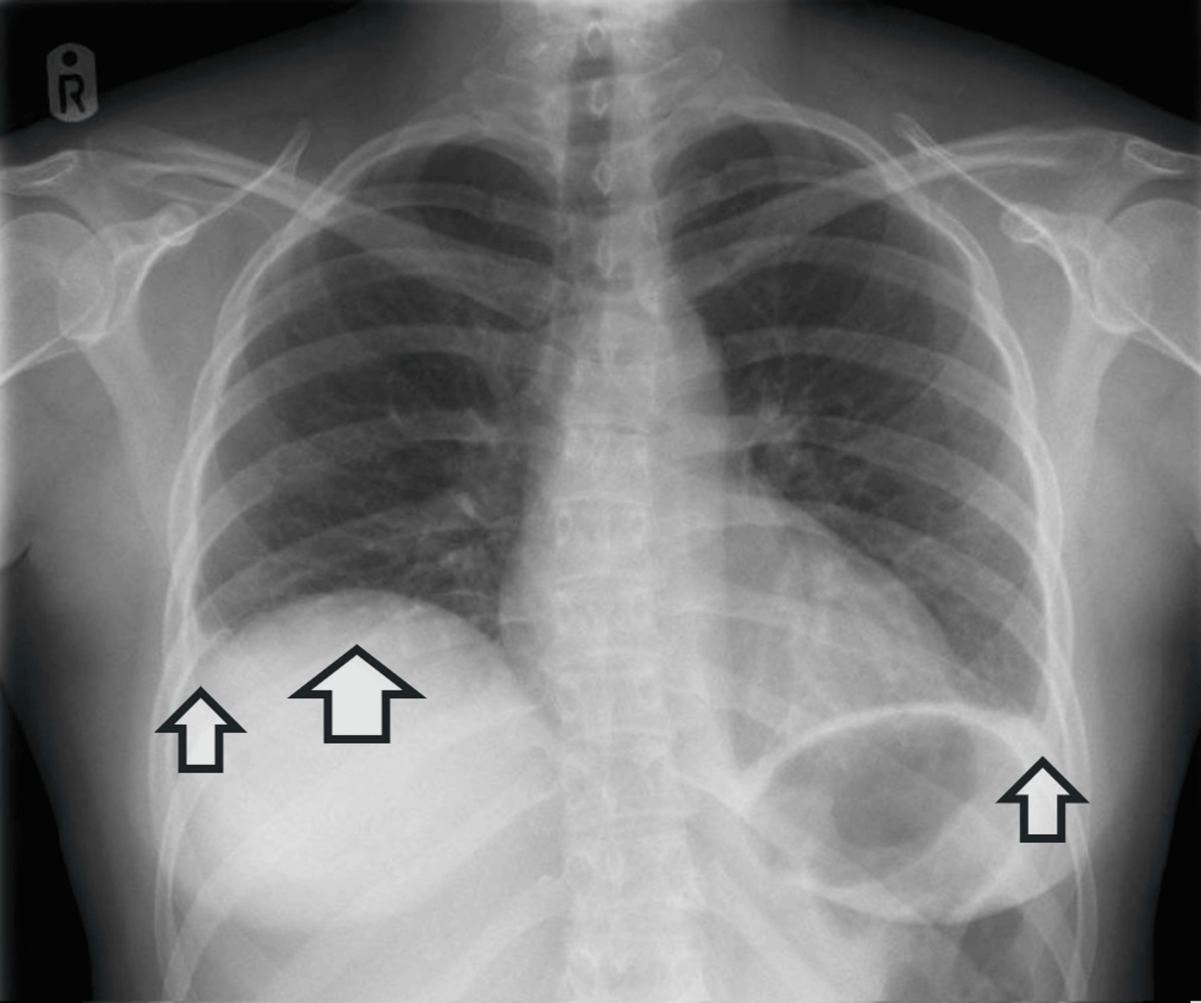
Chest X-ray showing raised right hemidiaphragm with bilateral small pleural effusions. Larger arrow denotes raised right hemidiaphragm, and smaller arrows denote bilateral small pleural effusions

Given the bilaterally reduced breath sounds, a chest radiograph was requested to exclude post-inflammatory pleural effusion. The image of the chest X-ray is shown in Figure 1, demonstrating a raised right hemidiaphragm and bilateral small pleural effusions.

**Figure 2:**
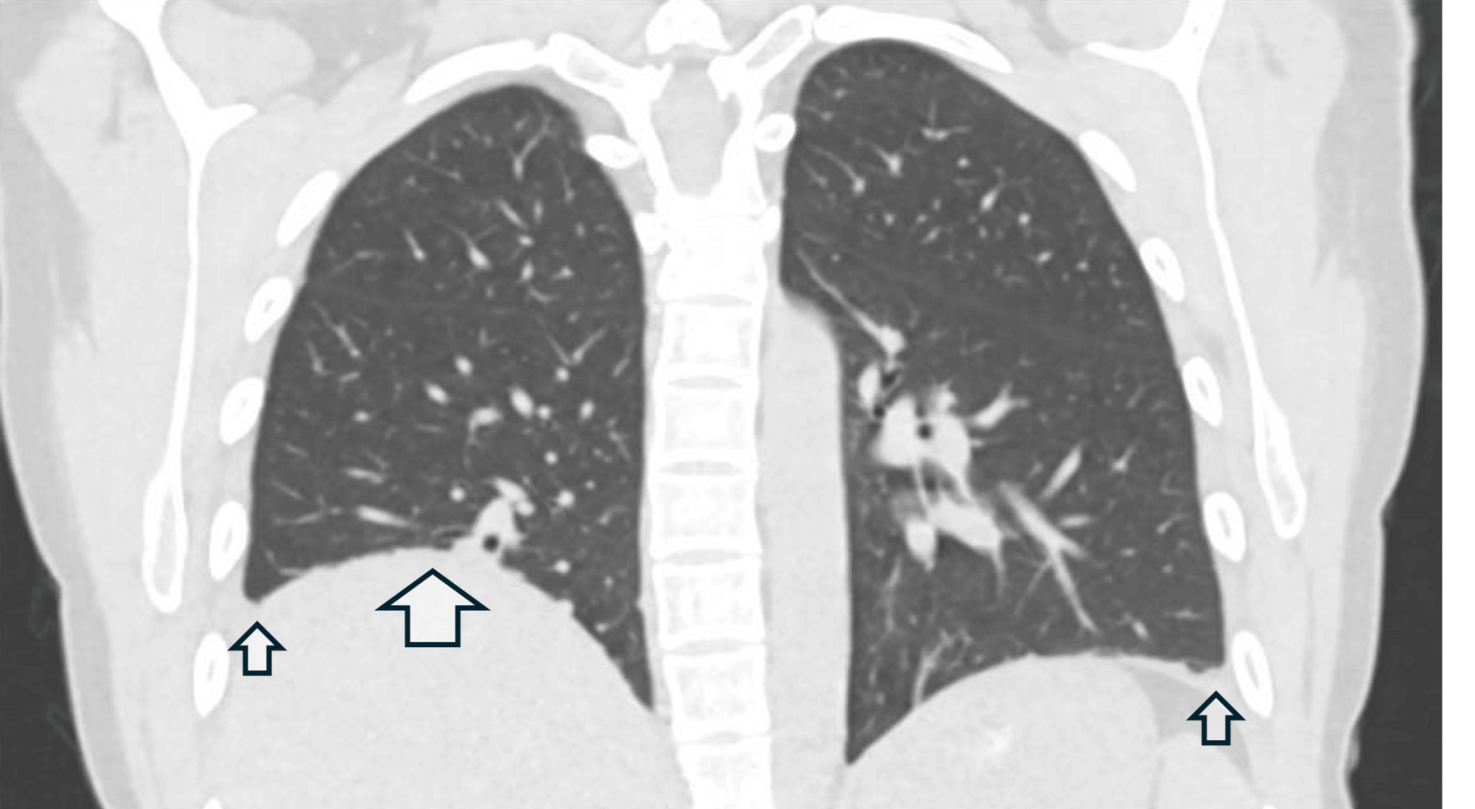
HRCT of the chest showing raised right hemidiaphragm and bilateral small effusions with otherwise normal lung parenchyma. The larger arrow shows a raised right hemidiaphragm, whereas the smaller arrows show small pleural effusions bilaterally. HRCT: high-resolution computed tomography

As symptoms persisted, a high-resolution computed tomography (HRCT) scan of the chest was also performed, which showed a persistently raised right hemidiaphragm with minimal atelectasis, along with trace pleural effusions bilaterally. It showed an otherwise normal lung parenchyma with no evidence of interstitial lung disease. Figure 2 shows a coronal view of her HRCT.

She was referred for pulmonary function tests, which confirmed a restrictive picture with reduced forced vital capacity (FVC), 34.3% of predicted, and a preserved FEV/FVC ratio of 1.12 (112%). There was a reduction in total lung capacity (TLC), 48% of predicted, and reduced gas transfer (TLCO), 51.4% of predicted. The relevant results of her pulmonary function tests are shown in Table 2.

**Table 2:** Initial lung function tests showing restrictive deficit, reduced TLC and gas transfer (TLCO). The standard ratio is a statistical measure that compares a patient’s lung-function test result with the predicted average value for a healthy population of the same age, sex, height, and ethnicity. FEV1: forced expiratory volume in 1 second; FVC: forced vital capacity; TLC: total lung capacity; TLCO: transfer factor of the lung for carbon monoxide

Variables	Predicted lower limit (in liters)	Actual (in liters)	% Predicted	Standard ratio
FEV1	3.04	1.45	38.4	-4.95
FVC	3.50	1.50	34.3	-5.72
TLC	4.63	2.70	48.0	-4.86
TLCO	6.59	4.36	51.4	-4.61

A neurological cause of raised hemidiaphragm and restrictive lung physiology was not considered likely, as she had a normal neurological examination. There were also no alarming symptoms reported by the patient, such as weakness, swallowing or speech disturbances, double vision, or a history of spinal or head trauma.

Her immune profile remained unchanged on subsequent follow-ups. However, considering the development of polyarthritis since her initial presentation, her diagnosis of MCTD evolved to SLE as per EULAR/ACR 2019 criteria [5]. In the context of her autoimmune illness and respiratory symptoms, coupled with restrictive lung disease and an elevated hemidiaphragm, she was diagnosed with shrinking lung syndrome.

The first phase of her treatment was immunosuppression with cyclophosphamide and prednisolone over the first three months. She was given prednisolone 15 mg once daily along with a total of six intravenous infusions of cyclophosphamide 800 mg. The first three cyclophosphamide infusions were given at two-weekly intervals, followed by the next three at three-weekly intervals. She was given prophylactic co-trimoxazole 960 mg once daily while on cyclophosphamide. During this phase of three months, her usual methotrexate (20 mg once weekly) was withheld while hydroxychloroquine (200 mg once daily) was continued. The rationale behind holding her methotrexate was to reduce the possibility of liver dysfunction while on cyclophosphamide.

**Figure 3:**
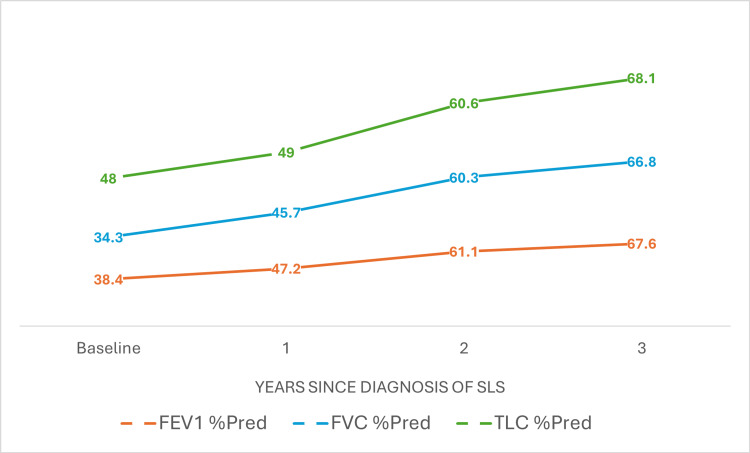
Serial lung function tests done at baseline and over time on a yearly basis since diagnosis of SLS showed an improvement in all parameters. FEV1: forced expiratory volume in 1 second; FVC: forced vital capacity; TLC: total lung capacity; % Pred: percentage predicted; SLS: shrinking lung syndrome

The second phase of her treatment was maintenance with rituximab, which was started a month after completing her course of cyclophosphamide. Two 1 g infusions of rituximab were given two weeks apart every six months for two years. After two years, rituximab was continued as an annual 1 g infusion. Alongside the rituximab, she was recommenced on her usual maintenance oral methotrexate 20 mg once weekly, and continued with hydroxychloroquine 200 mg once daily. After starting rituximab, her oral prednisolone was weaned down by 2.5 mg every month until it was eventually stopped. In addition to immunosuppression, she was advised to incorporate regular aerobic exercise in her daily routine to help improve her exercise tolerance. Over the course of treatment, she reported subjective improvement in her clinical symptoms compared to her initial presentation. Over the next three years, there was a significant improvement in her forced vital capacity from 34.3% to 66.8%, and a marginal increase in lung volumes from 48% to almost 68.1%. Figure 3 shows improvements in all parameters of serial lung function tests performed on a yearly basis since the diagnosis of SLS.

At the time of this report, she remains on extended interval annual rituximab infusions, along with oral methotrexate 20 mg once weekly and hydroxychloroquine 200 mg once daily.

## Discussion

The time of onset of SLS as a respiratory complication of SLE is variable, with SLS occurring at any point during the course of the disease. The average age of onset of SLS is four years, but it can also occur as a presenting feature of SLE [6]. Our patient developed symptoms of SLS at around the same time as her diagnosis evolved from MCTD to SLE.

Diagnosis of SLS is usually made on the basis of characteristic features of this condition (dyspnea, chest pain, reduced lung volumes, restrictive lung physiology) and the exclusion of other differential diagnoses. Therefore, laboratory investigations, imaging, and pulmonary function tests play a vital role in the evaluation of these patients. A positive ANA is seen in virtually all patients with SLS; however, it is non-specific. Other blood tests that may support the diagnosis of the underlying connective tissue disease causing SLS include antiphospholipid serology, hypocomplementemia, and a positive ENA test (including SS-A, SS-B, anti-RNP, and anti-Sm) [3]. However, laboratory investigations alone do not confirm the diagnosis. Chest radiography commonly shows unilaterally or bilaterally raised hemidiaphragms, often with reduced lung volumes. CT imaging is useful to rule out the more common differentials, such as interstitial lung disease or pulmonary emboli [7]. Lung function tests typically show a restrictive pattern of lung disease [4]. However, it must be reiterated that SLS remains a diagnosis of exclusion.

There are no standardized guidelines for the management of SLS, and although immunosuppression is the mainstay of treatment, the efficacy of various immunosuppressants remains poorly established. Corticosteroids are usually employed as first-line treatment, although the dosage and duration have not been established in any randomized controlled trials to date. It is most commonly dosed at 0.5-1 mg/kg of prednisolone daily, and carefully adjusted depending on individual patient response [3]. In more refractory cases, steroids are used in adjunct with other immunosuppressive agents such as cyclophosphamide, azathioprine, rituximab, belimumab, or mycophenolate mofetil [4,6,8]. Rituximab appears to be emerging as an effective drug for inducing remission in SLS and was found to be effective in our patient as well [4,9,10-12].

In Table 3 below, we compare our patient’s case with other published cases of SLS occurring in the setting of SLE. We can note that biologic agents, particularly rituximab, have been increasingly used as a treatment option in the last decade, with positive clinical outcomes.

**Table 3:** Comparison of our case with other published cases of SLS; rituximab is increasingly being used with good effect. SLS: shrinking lung syndrome; ANA: anti-nuclear antibodies; dsDNA: double-stranded DNA; RNP: ribonucleoprotein; C3: complement component; C4: complement component; TLCO: transfer factor of the lung for carbon monoxide

Studies	Age (years)/sex	Clinical features of SLS	Antibody profile	Chest imaging	Lung function tests	Treatment	Outcomes
Chaudhry et al., 2025 (present case)	20, female	Dyspnea, pleuritic chest pain	ANA, dsDNA, RNP, anti-Smith, Ro62	Raised right hemidiaphragm, small bilateral pleural effusions, normal lung parenchyma	Restrictive pattern, reduced TLCO	Cyclophosphamide, rituximab	Clinical improvement with improved lung function tests
Roostaei et al., 2025 [6]	33, female	Dyspnea, pleuritic chest pain	ANA, dsDNA, anti-phospholipid IgM, reduced C3 and C4	Raised right hemidiaphragm	Restrictive pattern, reduced TLCO	Corticosteroids, mycophenolate mofetil	Clinical improvement
Al-Karaja et al., 2023 [9]	35, female	Dyspnea, pleuritic chest pain	Not available	Bilateral basal atelectasis	Restrictive pattern	Corticosteroids, rituximab	Clinical improvement with improved lung function tests
He et al., 2024 [8]	35, female	Dyspnea, chest pain, fever, cough	ANA, dsDNA, anti-Smith, reduced complement	Thickened right pleura on CT	Restrictive pattern, reduced TLCO	Corticosteroids, tofacitinib	Clinical improvement with improved lung function tests
Shah et al., 2024 [11]	38, female	Dyspnea, pleuritic chest pain, non-productive cough	ANA, anti-cardiolipin, RNP, beta-2 glycoprotein, anti-Smith, reduced C3	Raised right hemidiaphragm	Restrictive pattern, reduced TLCO	Intravenous corticosteroids, rituximab	Clinical improvement
DeCoste et al., 2021 [12]	11, male	Dyspnea, pleuritic chest pain, non-productive cough	ANA, dsDNA, Ro, anti-Smith, reduced C3	Reduced lung volumes on HRCT, normal lung parenchyma	Restrictive pattern	Intravenous corticosteroids, rituximab	Clinical improvement
Goswami et al., 2016 [13]	38, female	Dyspnea, fever	ANA, Ro52	Elevated hemidiaphragms bilaterally	Restrictive pattern	Intravenous corticosteroids, rituximab	Clinical improvement, normalization of lung function tests

While most patients show an improvement in lung function tests with appropriate treatment, a study evaluating 35 cases of SLS showed that only 20% of the patients were able to achieve a normal vital capacity [8]. It is common for this condition to cause a chronic, mild restrictive deficit despite treatment. Untoward consequences such as respiratory failure, requirement of long-term oxygen, and death from this condition are extremely unlikely with this mild restrictive deficit [4].

## Conclusions

Shrinking lung syndrome remains a diagnostic dilemma in the setting of systemic autoimmune diseases, in particular SLE. The present case demonstrates that it can manifest as a pulmonary complication earlier on in the course of the disease. It is imperative to consider SLS as a cause of unexplained dyspnea in a patient with SLE. Timely diagnosis and initiation of appropriate immunosuppression should remain a priority as improvements in restrictive lung deficit and quality of life are achievable goals in the management of SLS. It should, however, be noted that a degree of chronic restrictive lung deficit can be expected in SLS despite treatment. Finally, this study underscores the need for standardized guidelines in the treatment and management of SLS. In particular, the safety and efficacy of biologic agents need to be studied as several reports have demonstrated their clinical benefit in patients with SLS. 
